# Analysis of Nasal Morphology in Skeletal Class I and Skeletal Class II Malocclusion: An Observational Study

**DOI:** 10.7759/cureus.29584

**Published:** 2022-09-26

**Authors:** Shruti Rathi, Rizwan Gilani, Ranjit Kamble, Rozina Vishnani

**Affiliations:** 1 Department of Orthodontics and Dentofacial Orthopedics, Sharad Pawar Dental College, Datta Meghe Institute of Medical Sciences, Wardha, IND; 2 Department of Orthodontics, Sharad Pawar Dental College, Datta Meghe Institute of Medical Sciences, Wardha, IND; 3 Department of Oral and Maxillofacial Surgery, Sharad Pawar Dental College, Datta Meghe Institute of Medical Sciences, Wardha, IND

**Keywords:** malocclusion, vertical pattern, class ii malocclusion, class i malocclusion, nasal morphology

## Abstract

Background and objectives

The nose is one of the major focuses of face attractiveness. Through careful evaluation of the soft tissue drape, a treatment plan can be designed to enhance a patient's facial attractiveness. The aim of this study was to evaluate and assess the variations in nasal morphology among class I malocclusion and class II horizontal and class II vertical malocclusion.

Material and method

Lateral cephalograms of 27 patients were taken and consisted of three groups: skeletal class I malocclusion, class II horizontal malocclusion, and class II vertical malocclusion. The various linear and angular measurements specific to nose were assessed.

Results and conclusion

In class II and class I malocclusion, the nose is observed to be straight and convex, respectively. Additionally, compared to class II horizontal malocclusion, it is more convex in class II vertical malocclusion. Vertical growers or high-angle cases are more likely to have a nose with an increased inclination toward nasal dorsum convexity than horizontal growers or low-angle instances.

## Introduction

Facial appearance is crucial to our rapid yet thorough impression of another person. For decades, anthropologists and physicians have attempted to objectively grasp the true meaning of facial beauty. Some physicians consider the nose to be the keystone of facial aesthetics since it is such a conspicuous aspect of the human face [1]. Czamecki, Nanda, and Currier reported that the shape of the nose and its relationship with other elements of the soft tissue profile influence people's perceptions of and attitudes toward face attractiveness [2]. As a result, a thorough examination of the nasal form and its relationship with other facial structures should be part of any patient evaluation prior to orthognathic surgery, rhinoplasty, or orthodontics.

Nasal development is thought to be steady throughout adolescence, peaking around the ages of 16 and 18 years for girls and boys, respectively [2]. In a lateral view, the ideal nasal proportion includes a straight nasal dorsum, with the supratip break created by the nasal-tip cartilage and dorsal cartilage above the nasal tip, and the alar rims 1 mm to 2 mm higher than the columella [ 3-5]. Nonetheless, nasal features like many other face characteristics differ from race to race [6-10]. The form of the nose can be used to determine age, gender, race, and ethnicity [11].

Some forms of treatment can change the shape of the nose and consequently the appearance of the face, either directly or indirectly. For example, orthodontic treatment for protruding incisors might result in lip alterations that raise the nose's relative prominence [12]. Because of alterations in the soft tissue chin and lower lip, mandibular surgery may affect the relative prominence of the nose. Maxillary and nasal surgery, on the other hand, are more likely to have a direct impact on the shape of the nose. Due to the lack of growth potential in adult skeletal class II patients, growth modification procedures such as functional jaw orthopedics are not possible. The initial choice for these patients is usually skeletal structure intervention in conjunction with orthodontics and surgery, known as orthognathic surgery. Camouflage treatment, on the other hand, entails hiding the underlying skeletal problem by shifting the maxillary dentoalveolar components and correcting occlusion. There are a variety of measurements to express the form and position of the nose and its relative position to facial structures.

This study included the Vidharbhian population with an objective to determine the value that defines a particular nasal appearance of an individual in skeletal class I and skeletal class II malocclusion with no specification of race, gender, and ethnicity. No such study has been conducted in this particular population earlier.

## Materials and methods

The individuals in this study were selected from the Department of Orthodontics, Sharad Pawar Dental College, Datta Meghe Institute of Medical Sciences, and the study was approved by the ethical committee (Ref. No.: DMIMS(DU)/IEC/2020-21/258). Only individuals who had no history of facial trauma, facial surgery, or orthodontic treatment were included.

The sample size was calculated using single proportion and was calculated to be nine in each group. This calculator uses the following formula for the sample size (n):

n=N*X(X+N-1),

where X=Za/22*p*(1-p)/MOE2 and Za/2 is the critical value of the normal distribution at a/2 (e.g., for a coincidence level of 95%, a is 0.05 and the critical value is 1.96), MOE is the margin of error, p= 0.95% is the sample proportion, and N is the population size.

A total of 27 samples were taken and divided into three groups based on the type of malocclusion and growth pattern, where ANB angle with 2° was class I malocclusion and ANB angle within a range of 4° to 6° was class II malocclusion horizontal growth pattern and class II malocclusion vertical growth pattern. The horizontal and vertical growth pattern was divided based on mandibular plane angle. The teeth were occluded in the intercuspal position, both lips were relaxed, and the head was kept in the natural head posture for the lateral cephalometric radiographs. The parameters studied in this study are specific to nasal structures, and soft tissue parameters that affect the nose are also considered in this study.

**Figure 1:**
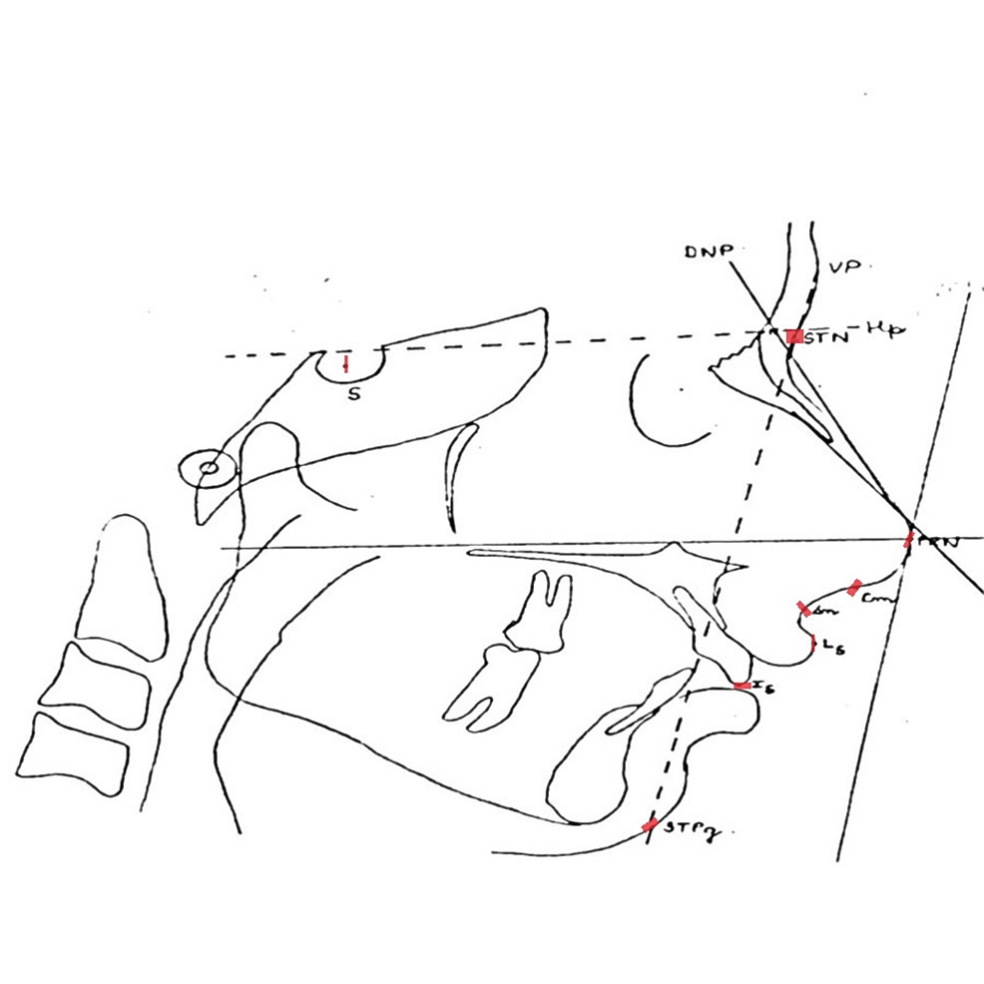
Cephalometric landmarks S, sella turcica; DNP, dorsal nasal plane is constructed by laying a straight edge on the upper aspect of the nose; STN, soft tissue nasion; Hp, horizontal reference plane constructed by drawing a line through soft tissue nasion parallel to the line through nasion 7° up from sella-nasion line; PRN, pronasale; Cm, columella, Sn, subnasale; Ls, labarale superius; Is, incision superius; STPg, soft tissue pogonion; VP, vertical plane The line drawn from VP to STPg is used for the horizontal measurements.

**Figure 2:**
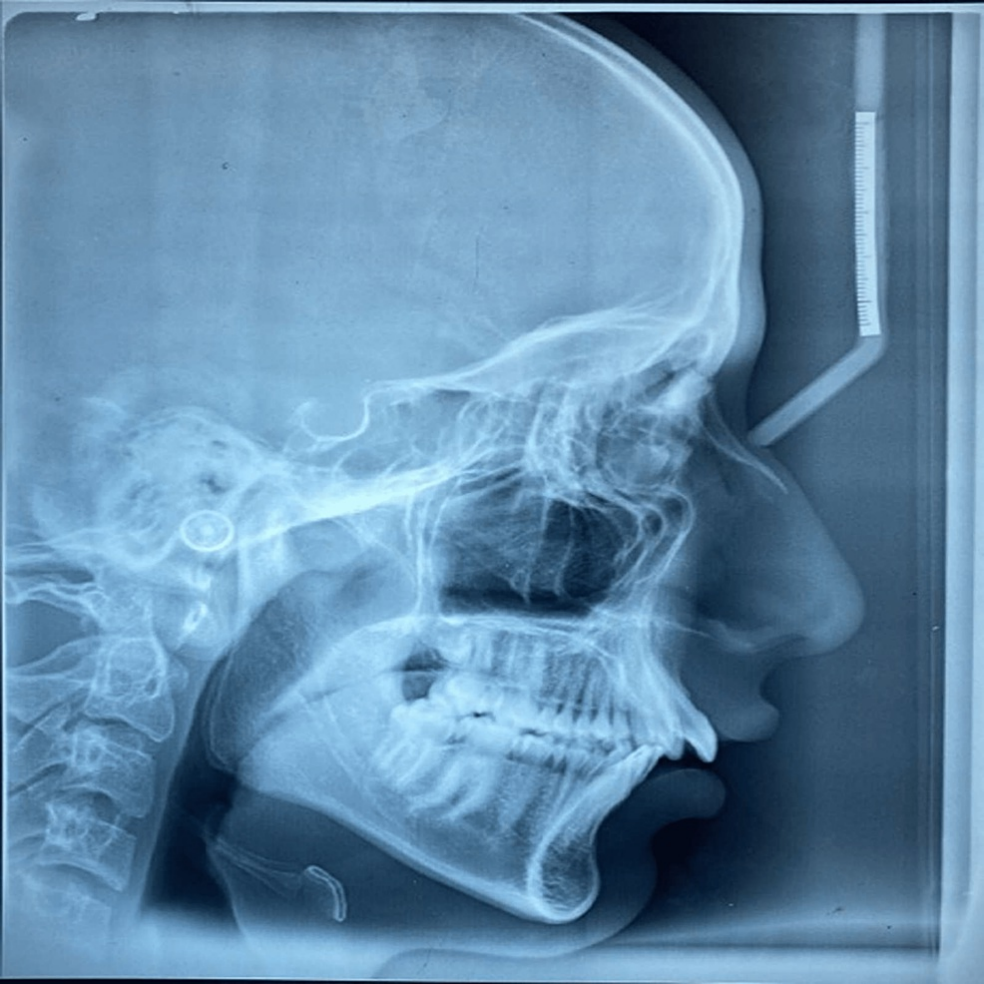
Lateral cephalogram This lateral cephalogram is used for tracing with the help of which the nasal linear and angular measurements will be measured.

On matte acetate tracing film, the reference point and planes listed in Table 1 were marked, and the second author checked them (Figure 1). Lateral cephalometric radiographs of each individual were used to determine the nose's size, shape, and location in relation to the other facial components (Figure 2).

**Table 1:** Reference points and planes This table depicts the description of the nasal landmarks used in this study.

Landmark	Definition
Cm	Columella point, the most anterior point on the columella of the nose [13]
DNP	Dorsal nose plane, constructed by laying a straight edge on the upper aspect of the nose
HP	Horizontal reference plane, constructed by drawing a line through soft tissue nasion parallel to the line through nasion 7° up from sella-nasion line
Is	Incision superius, the incisal edge of the most prominent maxillary central incisor
Ls	Labrale superius, the most anterior point on the upper lip
N	Nasion, the most anterior point of the nasofrontal suture in the midsagital plane
PRN	Pronasale, the most anterior point on nose [14]
S	Sella, the center of the pituitary fossa
STG	Soft tissue glabella
STN	Soft tissue nasion
STPg	Soft tissue pogonion
Sn	Subnasale
STP	Supratip plane
VP	Vertical plane

Further the linear and angular measurements were taken as described in Table 2.

**Table 2:** Linear and angular measurements This table depicts the various linear and angular measurements which are measured using the specific nasal landmarks. DNP, dorsal nasal plane is constructed by laying a straight edge on the upper aspect of the nose; STN, soft tissue nasion; Hp, horizontal reference plane constructed by drawing a line through soft tissue nasion parallel to the line through nasion 7° up from the sella-nasion line; PRN, pronasale; Cm, columella, Sn, subnasale; Ls, labarale superius; Is, incision superius; STPg, soft tissue pogonion; VP, vertical plane

Linear measurements	Angular measurements
STN-Sn nasal height	STG-STN-DNP nasofrontal angle
STN-PRN nasal length	STP-DNP supratip break angle
PRN-VP nasal depth	Cm-Sn-Ls nasolabial angle
PRN-Ls horizontal	Hp-DNP nasal projection angle
PRN-Ls vertical	STG-STPg-DNP nasofacial angle
PRN-STPg horizontal	-
PRN-STPg vertical	-
PRN-Is horizontal	-
PRN-Is vertical	-

## Results

By using the one-way ANOVA test, statistically significant results were found, as seen in Table 3.

**Table 3:** Results PRN, pronasale; Ls, Labarale superius; Is, incision superius; STPg, soft tissue pogonion

Measurement	Class I malocclusion		Class II horizontal malocclusion		Class II vertical malocclusion	
	Mean	SD	Mean	SD	Mean	SD
Nasal height	58.33mm	1.58	54.22mm	1.56	60.44mm	1.42
Nasal length	48.33mm	1.41	34.22mm	1.39	51mm	0.86
Nasal depth	27.11mm	1.61	20.44mm	1.13	27.88mm	0.78
Nasofrontal angle	135.77°	0.97	134°	3.14	139.11°	0.78
Nasolabial angle	111.66°	1.65	91.66°	1.22	113.77°	0.83
Nasal projection angle	126.11°	1.05	128.88°	1.05	131.22°	0.83
Supratip break angle	7°	1.22	10.88°	1.05	13.77°	0.66
Nasofacial angle	37°	0.86	39.77°	1.48	42.66°	1
PRN-Ls vertical	31mm	0.86	24.33mm	1.58	31mm	0.86
PRN-Ls horizontal	14.33mm	1.0	12.44mm	1.66	19.11mm	0.92
PRN-Is vertical	28.77mm	0.83	24.77mm	1.56	21mm	0.86
PRN-Is horizontal	41mm	0.92	44.66mm	0.92	38.22mm	0.83
PRN-STPg horizontal	19.33mm	1	16.33mm	1.22	22.44mm	0.88
PRN-STPg vertical	70.77mm	0.83	66.55mm	1.66	74.22mm	0.66

Nasal size

It was observed that the nasal height, length, and depth were significantly more in skeletal class II vertical malocclusion when compared to class I malocclusion and skeletal class II horizontal malocclusion. On comparing mean in the three groups, a statistically significant difference was found between class I and class II horizontal group (p=0.0001), between class I and class II vertical group (p=0.019), and between class II horizontal and class II vertical group (p=0.0001).

Nasal shape

It was observed that the supratip break angle was significantly more in skeletal class II vertical malocclusion when compared to class I malocclusion and skeletal class II horizontal malocclusion indicating that class I malocclusion has a straighter nose. On comparing mean in the three groups, no statistically significant difference was found between class I and class II horizontal group (p=0.0001), between class I and class II vertical group (p=0.0001), and between class II horizontal and class II vertical group (p=0.0001).

Nasal position

The individuals with skeletal class II vertical malocclusion had their noses significantly further from upper lips. On comparing mean in the three groups, a statistically significant difference was found between class I and class II horizontal group (p=0.0001), between class I and class II vertical group (p=0.1), and between class II horizontal and class II vertical group (p=0.0001).

## Discussion

The main aim of orthodontic therapy is to improve facial appearance. The nose has a significant impact on the aesthetics of the face because it is the main feature of the human face. The aesthetics of the facial profile are greatly impacted by the variance in the form, shape, and size of the nose. The anterior teeth should be carefully positioned by orthodontists to avoid the nose appearing relatively prominent. Due to the chance that the nose or other face structures may have been impacted, the study excluded participants with a history of facial surgery, facial fractures, or orthodontic therapy.

The literature suggests no correlation between a person's skeletal class and their amount of nasal growth [14]. Chaconas reported that class I subjects mainly had a straighter nose and class II subjects had a convex nose [15].

The nasal depth is found to be least in class II horizontal malocclusion, which indicates less prominence of the nose. Class II vertical malocclusion has a prominent nose; therefore, the treatment care must be formulated according to the profile view of an individual. The nasofrontal angle in class II vertical malocclusion has significantly more value. This study demonstrated that the mean supratip break angle was found to be 13.77° in class II vertical malocclusion as compared to 7° and 10.8° in class I and class II horizontal malocclusion, respectively. This study demonstrated that the mean nasofacial angle was found to be 42.66° in class II vertical malocclusion as compared to 37° and 39.77° in class I and class II horizontal malocclusion, respectively. It has been seen that the nasolabial angle is lesser in class II horizontal malocclusion when compared to class I and class II vertical malocclusion.

The nasal projection angle showed no significant difference in all the three groups. In the present study, the vertical distance from the pronasale to upper incisor and soft tissue pogonion was less in class II horizontal malocclusion when compared to class I and class vertical malocclusion. Also, it was observed that the vertical distance from the pronasale to lower incisor was least in class II vertical malocclusion. The horizontal distance from the pronasale to lower incisor and soft tissue pogonion was least in class II horizontal malocclusion, along with the horizontal distance from the pronasale to upper incisor.

The current study's contribution is the establishment of nasal standards that can be considered as a guide during the diagnostic and treatment planning of patients undergoing orthodontic treatment, orthognathic surgery, and rhinoplasty, hence improving the results following treatment, especially in the population of Vidharbh. Future research should take into account a larger sample size of each gender as there was no comparison in terms of gender.

## Conclusions

A long nose with enhanced nasal prominence (nasal depth) is expected in people with long faces as well as those with long upper and lower jaws, according to the assessment of the most frequent nasal parameters from this study. A short/normal face with short/normal upper and lower jaws is predicted to have a short/normal nose with short/normal nasal depth. In class II and class I malocclusion, the nose is observed to be straight and convex, respectively. In class II vertical malocclusion as opposed to class II horizontal malocclusion, it is also more convex. Vertical growers or high-angle cases are more likely to have a nose with an increased inclination toward nasal dorsum convexity than horizontal growers or low-angle instances. As this study was conducted in the Vidharbhian population, it can act as a guide in the treatment planning of the individuals of this population.
